# New insights into antibody levels against SARS-CoV-2 for healthcare personnel vaccinated with tozinameran (Comirnaty)

**DOI:** 10.1371/journal.pone.0276968

**Published:** 2022-11-03

**Authors:** Antonio Fernández-Suárez, Rosa Jiménez Coronado, Carlos Clavijo Aroca, Estrella Navarro Martín, Amir Qmega Qmega, José Miguel Díaz-Iglesias

**Affiliations:** 1 Department of Biotechnology, Alto Guadalquivir Hospital, Andújar, Jaén, Spain; 2 Department of Pathology, Alto Guadalquivir Hospital, Andújar, Jaén, Spain; Stanford University School of Medicine, UNITED STATES

## Abstract

**Aim:**

The aim of this study is to determine the levels of spike protein IgG and total antibodies in subjects vaccinated against SARS-CoV-2 (both infected and non-infected) and the titer evolution over time. In addition, we also addressed the performance of each of the included platforms in the study, as they are intended to measure antibody levels in naturally infected patients.

**Materials and methods:**

An observational study including 288 volunteer healthcare professionals vaccinated against SARS-CoV-2 (Comirnaty^™^) at the Andújar Alto Guadalquivir Hospital. Serum samples were obtained in September 2020 and 14 and 90 days after administration of the second dose. The following *in vitro* methods were used: Elecsys Anti‐SARS‐CoV‐2 N and Elecsys Anti-SARS-CoV-2 S (Roche, Germany) and EliA SARS-CoV-2-Sp1 IgG (Thermo Fisher Scientific, Germany).

**Results:**

For the Elecsys S method at 1/10 dilution and for the EliA Sp1 IgG method at 1/5 dilution, 54% and 19% of samples were out of range, respectively. The vaccine activated a high humoral response– 0 to 3000 BAU/mL being the “normal titer range” in all volunteers. Patients vaccinated after COVID-19 exhibited higher total S antibody load values than non-vaccinated volunteers while showing the same response for S IgG isotype. Titers decreased up to 86% in the case of S IgG neutralizing antibodies.

**Conclusions:**

The characterization of human response to SARS-CoV-2 vaccines is still far from being completely elucidated. It is important to increase the methods dynamic range to study humoral response evolution in depth and decide whether booster doses or seasonal vaccination plans will be necessary to definitively control the pandemic.

## Introduction

The novel SARS-CoV-2 was first described in the Chinese city of Wuhan in late 2019 and rapidly spread worldwide [1]. On March 11, 2020, the World Health Organization declared the COVID-19 disease a pandemic [1], which is still a global threat two years and a half later. There have been 626,985.198 cases of SARS-CoV-2 infection and 6,576.037 [2] death around the world as of October 2022.

As no effective treatment has been approved for human use during the ongoing pandemic, vaccines are the most powerful weapon against the SARS-CoV-2 pandemic to reduce the morbidity and mortality of COVID-19 [3]. At the time this article was drafted, four vaccines had been approved in Europe by the European Medicines Agency: Comirnaty^™^ (Pfizer-BioNtech), Vaxzebria (AstraZeneca), COVID-19 vaccine (Moderna) and COVID-19 vaccine (Janssen) [4].

Of the four structural proteins of the infectious virion, spike protein (S), N protein (nucleocapsid), M protein (matrix), and E protein (envelop), only N and S have been shown to trigger the production of high antibody titers in naturally infected patients. However antibodies to N are unlikely to neutralize the virus [5]. For most COVID-19 vaccines produced, this is the reason why the outcome priority was antibody generation by T cells [6], specifically neutralizing antibodies against spike protein containing RBD, the receptor binding domain [5].

There are 200 serological techniques available worldwide to detect SARS-CoV-2 antibody isotypes (IgG, IgM, and IgA) as well as different combinations of them simultaneously [7]. According to the Interim Guidelines for COVID-19 antibody testing from the CDC (Centers for Disease Control and Prevention), serological tests can play an important role in supporting the diagnosis of acute COVID-19 illness and, as a complement to qPCR, to increase the sensitivity of the nucleic acid detection method [8], but they should not be used to establish or exclude SARS-CoV-2 infection or reinfection, neither for the establishment of immune status nor until immunity is fully characterized [9].

In all countries with ongoing vaccination plans at present, serological tests are now in the spotlight as a method to determine the efficacy of the vaccines. However, as most of the tests were intended for antibody testing in naturally infected volunteers, more data is needed for the use of the tests in vaccinated volunteers whose antibody levels are expected to be considerably higher, both if they have been previously infected or not. Also, further evidence is needed on the distribution of the antibody titers in vaccinated volunteers for each vaccine and the evolution of the antibody titers over time, which is required to determine the need for booster doses in the near future.

The aim of this study is to describe the distribution of total S and IgG S antibodies in two cohorts -SARS-CoV-2 infected and non-infected- vaccinated volunteers-, and the titer evolution over time. In addition, we will compare the results of two different testing platforms to describe the adjustments needed for each method for the appropriate measurement of the antibody load.

## Materials and methods

This observational study included 256 volunteer healthcare professionals vaccinated against SARS-CoV-2 (Comirnaty^™^) at the Andújar Alto Guadalquivir Hospital who had not been previously infected by SARS-CoV-2. All patients were tested for SARS-CoV-2 nucleoprotein antibodies to ensure they had not been infected previously (Table 1 in S1 File). Blood samples were taken to obtain three time point antibody values: September 2020 (baseline, before vaccination), 14 (immunization peak) and 90 (long-term immunization; not all volunteers, n = 27) days after the administration of the second dose of the vaccine. Serum samples were stored at -20ºC and, after finishing sera recruitment at each time point, all samples were measured in one run. Inclusion criteria: professionals >18 years old, with informed consent signed, full vaccination (two doses), and no previous infection (negative PCR and serological test and no evidence of the disease after an exhaustive review of the medical records of all volunteers).

To compare S IgG antibody titers from vaccinated and infected volunteers with non-vaccinated subjects, we retrospectively analyzed S IgG antibody titers from patients naturally infected (n = 60), who were tested using EliA method within 14 to 60 days after the positive PCR result.

The study also included a small cohort consisting of 32 vaccinated volunteers randomly selected who had been diagnosed with COVID-19 before September 2020 (same inclusion criteria as below, but positive PCR and mild to moderate clinical symptoms). Blood samples were obtained 14 days after the administration of the second dose and handled as described previously.

The study was approved by the Ethics Committee from Hospital Alto Guadalquivir and all enrolled volunteers signed the corresponding informed consent.

The demographic and laboratory clinical data were obtained from the INFINITY laboratory computer system (Roche, Germany) and exported to an anonymized file that was further completed with the serological results.

### Antibody measurement

Three *in vitro* diagnostic methods were used for the determination of antibodies against SARS-CoV-2:

- Elecsys Anti‐SARS‐CoV‐2 (Roche, Germany). ECLIA immunoassay for the qualitative *in vitro* detection of total antibodies (including IgG) against N protein.
- Elecsys Anti-SARS-CoV-2 S (Roche, Germany). Quantitative detection of spike protein antibodies (S) through the ECLIA technique. The Elecsys Anti-SARS‐CoV‐2 S assay uses a double-antigen sandwich assay. Determinations were performed using COBAS 8000 automated analytical platforms.
- EliA SARS-CoV-2-Sp1 IgG (Thermo Fisher Scientific, Germany). Quantitative detection of antibodies against the S1 subunit of the spike protein. The EliA SARS-CoV-2-Sp1 IgG test uses the EliA IgG method (fluoroenzyme immunoassay). Determinations were performed on the Phadia 250 instrument.

All tests were carried out in accordance with the manufacturer’s specifications. However, for some of the serum samples corresponding to vaccinated volunteers, antibody titers were out of the calibration range and samples needed to be diluted. For the Elecsys Anti-SARS-CoV-2 S method, all samples were diluted to 1/10 (manufacturer’s recommendation). However, some results were still out of range, so 1/20, 1/40, and 1/100 dilutions were needed to characterize all included samples. Due to the large number of samples processed requiring further dilutions for this method, an imprecision study was carried out following the EP05-A2 protocol guidelines of the Clinical and Laboratory Standards Institute (CLSI) using three different levels (S1 = 411.41 BAU/mL, S2 = 1,576.13 BAU/mL, S3 = 2,264.40 BAU/mL).

For the EliA SARS-CoV-2-Sp1 IgG method, all samples were diluted to 1/5 (manufacturer’s recommendation), and 1/10 and 1/20 dilution was required in a few cases.

### Data analysis

Demographics were expressed in terms of the total number of volunteers and percentages. Antibody titers obtained from analytical systems were transformed into BAU units (1 BAU = 0.972 Elecsys units and 0.25 EliA units), corresponding to the WHO International Standard and then classified according to the following BAU/mL ranges: 0–500; 500–1,000; 1,000–1,500; 1,500–2,000; 2,000–2,500; 2,500–3,000; 3,000–3,500; 3,500–4,000; 4,000–4,500; 4,500–5,000 and 5,000–10,000 in order to characterize titer distribution across the volunteer cohort.

For statistical purposes in both cases (EliA IgG S and Elecsys S), volunteers were then classified into two BAU/mL ranges: 0–3,000 and ≥3,000–10,000. A nonparametric test was used to determine the association between the antibody titer, age, and sex (Mann-Whitney test). Spearman’s correlation was used to assess monotonic relationships. All calculations were performed using SPSS 17.0 (SPSS Inc., Chicago, USA).

## Results

### 1. Demographics

Of the 256 non-infected enrolled volunteers 81% (n = 207) were women and 19% (n = 49) were men. The average age was 45 years old (range: 24–67; average age: 44 for women, 47 for men) (Table 1).

**Table 1:** Vaccinated volunteers’ demographic and serological data overview.

Volunteers	Sex	Age (years, average)	Elecsys S (BAU/mL) (median)	EliA Sp1 IgG (BAU/mL) (median)
Vaccinated, non-infected	80% women	45.5	1950.62	2380.00
Vaccinated, infected	84% women	39.0	2525.00	2456.00

The SARS-CoV-2 infected cohort included 84% (n = 27) of women and 16% (n = 5) of men. The average age was 39 years old (Table 1).

### 2. Test performance

Test performance analysis was carried out in non-infected patients. For the Elecsys S method at 1/10 dilution, 54% of the samples were out of range. For the 1/20 and 1/40 dilutions, there were also 12% and 0.4% of samples out of range, respectively, so a final dilution of 1/100 was needed to characterize all samples.

For the EliA IgG method at 1/5 dilution, 19% of the samples were out of range, 1% at 1/10 and a 1/20 dilution was sufficient to determine IgG levels in all sera.

### 3. Evaluation of Elecsys S assay precision

The variance coefficient was 1.34% and 1.30% for the two samples corresponding to 1/10 dilution. At a 1/20 dilution, the coefficient of variation increased to 4.92%.

### 4. Antibody titers

#### 4.1. SARS-CoV-2 uninfected vaccinated volunteers

In September 2020 all included volunteers were screened for total N antibodies. Results were negative for all of them with an average load of 0.09 U/mL, in agreement with clinical records from included volunteers that were carefully reviewed from September to the vaccination date without finding any evidence of infection in a highly controlled population (health care professionals).

**Fig 1:**
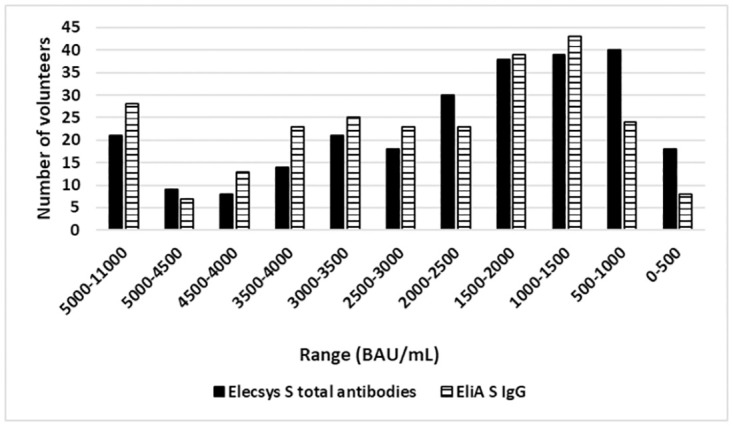
Distribution of the 256 non-infected volunteers through different IgG antibody ranges for both methods used. Volunteers were grouped according to their S protein total antibody and IgG titers (0–500, 500–1000,1000–1500,1500–2000, 2000–2500, 2500–3000, 3000–3500, 3500–4000, 4000–4500, 4500–500 and 5000–11000 BAU/mL).

For the Elecsys S method, the total antibody levels detected ranged from 55.81 to 10,648.15 S BAU/mL, with a median of 1,950.62 S BAU/mL. For the EliA IgG method, measuring only spike targeted IgG, values ranged from 62 to 9,320 BAU/mL S IgG with a median of 2,380 BAU/mL S IgG; although both methods (S) measure different parameters, titer distribution in the subjects was quite similar (Fig 1). Even though both platforms tested against spike protein, they had different methodological designs and detected different targets (Isotype mixture *vs*. IgG), so a correlation study was carried out. A Spearman’s Rho coefficient of 0.810 was obtained with significant differences (p <0.001).

Both for EliA and Elecsys, the most common values fell inside the 0 to 3,000 BAU/mL IgG/total range, including 63% of volunteers for EliA and 71% for Elecsys. 3,000 BAU/mL was selected as the cut-off value between the “common titers” group and the “super-responders’ group” as clear statistically significant differences were shown between them (p<0.001) (Table 2) for both methods. In both cases, the range from 500 to 2,000 BAU/mL represented the highest number of volunteers (Fig 1). Interestingly, values were considerably lower for volunteers naturally infected but unvaccinated, as expected 88% of those patients displayed titers that were under 800 BAU/mL (Fig 1 in S1 File).

**Table 2:** Descriptive analysis of antibodies concentrations by Elecsys Anti-SARS-CoV-2 S and EliA SARS-CoV-2-Sp1 IgG in the 256 non-infected volunteers included in the study.

	Elecsys S (BAU/mL)		EliA Sp1 IgG (BAU/mL)	
n (%)	Median (P_50_)	(P_25_—P_75_)	Median (P_50_)	(P_25_—P_75_)
**Total**	**1950.62**	**1095.68–3231.99**	**2380.00**	**1330.00–3640.00**
**256**				
Women	2097.74	1192.39–3403.81	2520.00	1450.00–3760.00
207 (80.9)				
Men	1316.87	809.77–2016.46	1620.00	1180.00–2680.00
49 (19.1)				
p value	<0.001*		0.004*	
**Cut-off High titers**	**<3000**	**≥3000**	**<3000**	**≥3000**
n (%)	183 (71.5)	73 (28.5)	160 (62.5)	96 (37.5)
Age (average)	45.49	43.62	46.15	42.96
Women,n	140	67	121	86
Men,n	43	6	39	10
Women/men ratio	3.25	11.16	3.10	8.60
Median (P_50_)	1469.14	4089.51	1540.00	3970.00
(P_25_-P_75_)	888.74–2023.66	3446.50–5089.51	1130.00–2190.0	3470.00–5140.00
p value	<0.001*		<0.001*	

P_25_: Percentile 25; P_75_: Percentile 75;

*significative differences (Mann-Whitney Test).

It is worth highlighting that for subjects with values greater than 3,000 BAU/mL, higher than the median titers, the female/male ratio was considerably higher than for the other volunteers with lower values. In fact, for Elecsys, the ratio of women to men was 3.3 for volunteers with titers up to 3,000 S BAU/mL and 11.2 for ≥ 3,000 to 10,000 S BAU/mL. Median antibody load values between men and women were also statistically different for the Elecsys method (p<0.001). For EliA, volunteers with titers from 0 to 3,000 BAU/mL S IgG had a ratio of women to men at 3.1 *vs*. 8.6 for higher range values. Similarly, in this case, median value differences were also statistically significant (Table 1; p = 0.004). Furthermore, there was only one case of a man with values above 5,000 BAU/mL for Elecsys and 4 for EliA, while 23 women for EliA and 20 for Elecsys exceeded that value.

The average age of volunteers in each of the ranges for both methods was almost the same and no statistical differences were shown for each group (Table 2).

**Fig 2:**
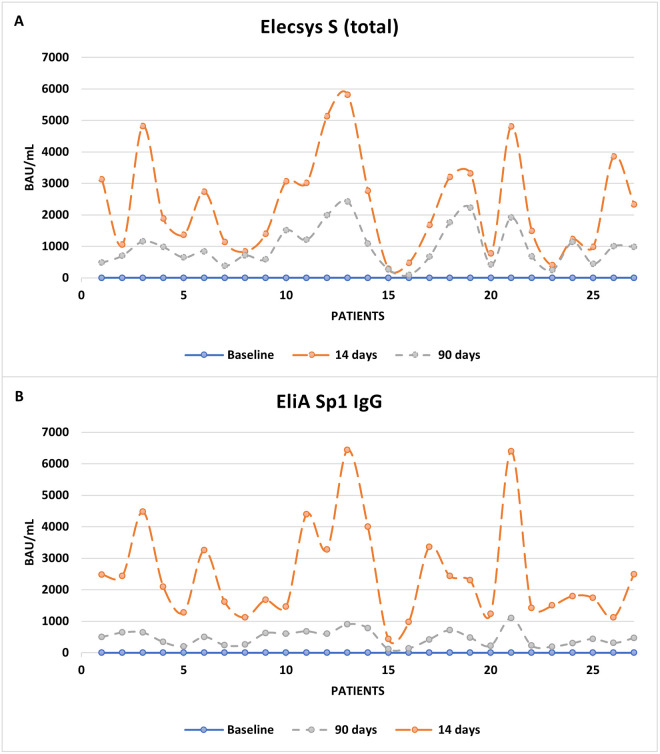
Antibody titer evolution at inclusion, 14 and 90 days after vaccination. **A.** S protein total antibodies. **B**. S protein IgG antibodies.

Three months after the vaccination, 27 of the 256 volunteers were randomly selected and retested. All of them had a marked decrease in antibody titers. S IgG showed an average titer decrease of 81% and total S antibodies of 51%. In both cases titer differences were statistically significant at 14 and 90 days after vaccination: Elecsys S (2,525.11±357.89 BAU/mL *vs*. 1,108.63±173.61 BAU/mL; p<0.001) and EliA Sp1 IgG (2,456.30±298.90 BAU/mL *vs*. 478.30±48.35 BAU/mL; p<0.001) (Fig 2).

### 4.2. SARS-CoV-2 infected patients

Antibody titers were also assessed in a small cohort of volunteers who had recovered from COVID-19 prior to vaccination (n = 32). For the Elecsys method, total antibody levels were significantly higher compared to the naïve volunteers: (p<0.001) 18545.89 BAU/mL (P25: 4,600.82—P75: 26,158.56) *vs*. 1,950.62 BAU/mL (P25: 1,092.08—P75: 3,239.97). For the EliA method, conversely, levels remained the same as for non-infected volunteers: 2,140.00 BAU/mL (P25: 1,680.00—P75:3,370.00) *vs*. 2,380.00 BAU/mL (P25: 1,330.00—P75: 3,640.00), p = 0.881.

## Discussion

A key point when analysing antibody titers and vaccine outcomes in the COVID-19 pandemic context is the knowledge of the patient infection status. Our results show that positivity to N specific antibodies is a reliable marker to differentiate vaccinated subjects who have already had the disease naturally from vaccine *naïve* subjects whose first and only contact with the viral antigens has been through the vaccine. In fact, some volunteers were excluded from the study due to COVID-19 clinical history, and in all cases the total antibodies against the nucleocapsid (N) were positive. This situation may be completely different for another type of vaccine, and thus the results found in this study should be limited only to Comirnaty^™^ or a vaccine with a similar design.

Our data reveals that the methods tested for the detection of antibodies against S protein are not adapted to the management of serology in vaccinated subjects, since in many cases the levels of antibodies are so high that they require dilutions to be quantified. This was especially relevant in the Elecsys S method, where 54% of the serum samples required higher dilutions (≥1/20) than the recommended one (1/10). However, the imprecision study carried out indicates that the results are quite reproducible even at these high concentrations of antibodies, although the coefficient of variation increases with higher dilutions. In contrast, the EliA method only required 19% of the samples to be diluted above the recommended range (1/5). In any case, in view of these findings, manufacturers must adapt their methods to be able to perform quantification in vaccinated subjects without needing to dilute serum samples. This is especially relevant when it is essential to make high dilutions that can increase analytical errors in processing. In any case, the imprecision in the methods caused by these high concentrations, and in view of the results found in the medium-term follow-up, would only occur in weeks very close to vaccination.

We found great heterogeneity in the immune response of the subjects after vaccination for both S antibody detection tested methods, particularly relevant for the Elecsys S method, where values ranged from 100 to 10,648 BAU/mL. The results found for S1 IgG antibodies in the EliA method were much more clustered. Nonetheless, a range of 0–3,000 BAU/mL would be appropriate to be established as common values for the vaccinated population.

We observed that most of the volunteers with high levels (≥3,000 BAU/mL) of spike protein antibodies were women. Even though many of the subjects included in the study are women, the female to male ratio was 3.5 times higher for Elecsys and 2.8 as high for EliA. This new finding should be further investigated in depth and could indicate that some unknown variable(s) associated with the female sex could cause a more intense immunization through this vaccine in a subgroup of women.

Our study of antibody levels 3 months after the complete immunization revealed important results. Antibody titers decreased in our population both at total and IgG level. Interestingly, the decline was homogeneous and sharper for S IgG while it was heterogeneous and less pronounced for S total antibodies, probably due to a higher decrease in IgG than the IgM and IgA isotypes load. The question rising from these results is whether humoral response will continue decreasing or not over time and thus whether a booster dose of Comirnaty^™^ will be needed or not. In any case, we must bear in mind that current vaccines also stimulate cellular immunity.

Finally, our small evaluation of the antibody levels in COVID-19 recovered volunteers revealed that the infection increased the humoral response to the vaccine in terms of total antibodies but not of IgG production. This behaviour needs to be studied in depth and also its consequences.

There are a few limitations inherent to the design and the subjects included in this study. First, the sample size is limited, especially for the COVID-19 infected cohort. Second, almost all volunteers fall under a similar age range. Finally, there is a predominance of females due to their greater presence in the healthcare sector.

## Conclusions

The levels of antibodies in vaccinated subjects are high and heterogeneous, so testing using commercial assays need to be fine-tuned to increase their dynamic ranges. A 0–3,000 BAU/mL range could be established as “common values”.

Comirnaty^**™**^ induces an immediate high humoral response, but titers considerably decrease during the first three months, especially for IgG isotype. Moreover, in COVID-19 infected patients the immune response induced by the vaccine is greater than the one induced by natural infection. The combination of infection and vaccine improves total S antibodies production but not IgG.

## Supporting information

S1 File (DOCX)

S1 Fig (TIF)
