# Supplementary figures and images for: New insights into antibody levels against SARS-CoV-2 for healthcare personnel vaccinated with tozinameran (Comirnaty)
Source: PLoS One. 2022 Nov 3;17(11):e0276968. doi: 10.1371/journal.pone.0276968 (PMC9632819; doi:10.1371/journal.pone.0276968)

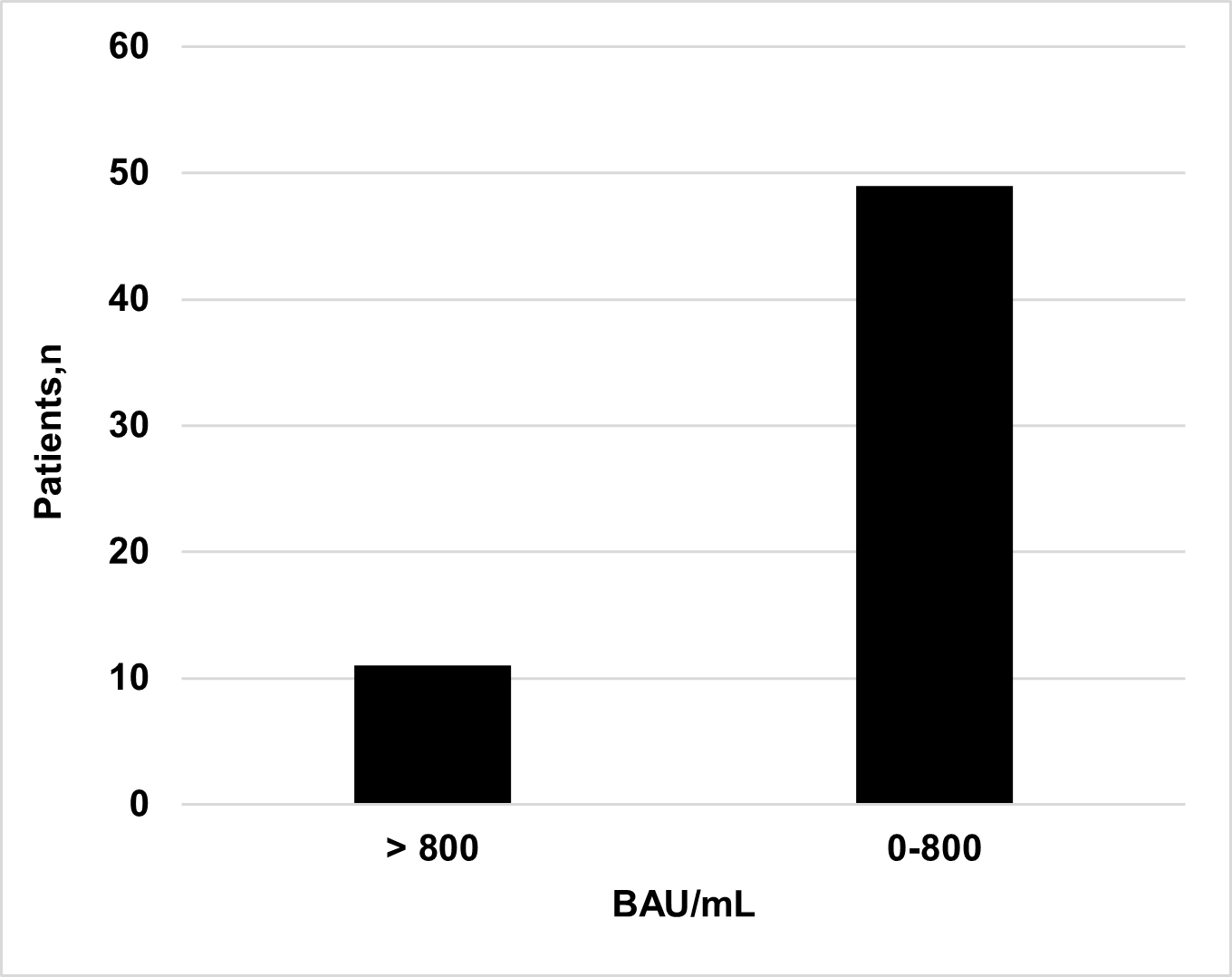

Supplement: S1 Fig — (TIF) [file pone.0276968.s002.tif]
